# Genetic variations associated with immediate hypersensitivity reactions to iodinated contrast media: A whole exome sequencing study

**DOI:** 10.1371/journal.pone.0345313

**Published:** 2026-03-26

**Authors:** Noeul Kang, Hoshik Kwon, Myung-Eui Seo, Byung-Joo Min, Byung-Jae Lee, Ju Han Kim, Ho Yun Lee

**Affiliations:** 1 Division of Allergy, Department of Medicine, Samsung Medical Center, Sungkyunkwan University School of Medicine, Seoul, Republic of Korea; 2 Department of Psychiatry, Asan Medical Center, University of Ulsan College of Medicine, Seoul, Republic of Korea; 3 Division of Biomedical Informatics, Seoul National University Biomedical Informatics (SNUBI), Seoul National University College of Medicine, Seoul, Republic of Korea; 4 National Forensic Service Seoul Institute, Seoul, Republic of Korea; 5 Department of Radiology and Center for Imaging Science, Samsung Medical Center, Sungkyunkwan University School of Medicine, Seoul, Republic of Korea; 6 Department of Health Sciences and Technology, SAIHST, Sungkyunkwan University, Seoul, Republic of Korea; Deccan School of Pharmacy, INDIA

## Abstract

**Objective:**

The use of iodinated contrast media (ICM) in computed tomography (CT) has increased significantly; however, hypersensitivity reactions (HSRs) remain a concern. This study aimed to investigate genetic factors associated with ICM-induced immediate HSRs using whole exome sequencing (WES).

**Materials and Methods:**

We conducted a case–control study including 20 patients with ICM-induced immediate HSRs and 11 controls who had received ICM at least three times without HSRs. WES was performed with DNA extracted from saliva samples. Analyses included single-nucleotide variant (SNV) association testing using the Cochran–Armitage trend test with false discovery rate (FDR) correction, gene-wise variant burden (GVB) analysis, and copy number variation (CNV) detection using complementary algorithms.

**Results:**

A variant in FAST kinase domain 1 (*FASTKD1*, rs12618227) was significantly more prevalent in the control group compared with the case group (72.7% vs. 5.0%, FDR *p* < 0.10), suggesting a protective role. GVB analysis revealed lower scores for *FASTKD1* and 2-hydroxyacyl-CoA lyase 1 (*HACL1*) in the control group (nominal *p* < 0.001). CNV analysis identified a significant Signal Regulatory Protein Beta 1 (*SIRPB1*) deletion in the case group (5/20, 25.0%). In contrast, CNVs in Mucin 12, cell surface associated (*MUC12*) were observed in both groups. Immune cell expression data showed high expression of *FASTKD1*, *HACL1*, and *SIRPB1* in granulocytes, particularly basophils.

**Conclusion:**

*FASTKD1* and *HACL1*, which are involved in mitochondrial and metabolic regulation, and *SIRPB1*, which participates in innate immune signaling, were identified as candidate genes potentially associated with ICM-induced immediate HSRs. These suggest a possible contribution of both metabolic and immune regulatory pathways to genetic susceptibility and require validation in larger, independent cohorts before clinical application.

## Introduction

Iodinated contrast media (ICM) are used with computed tomography (CT) to improve diagnostic accuracy in evaluating diseases and assessing treatment responses. More than 75 million CT scans using ICM are performed globally each year [1]. Although non-ionic ICMs have largely replaced monomeric ionic ICM due to fewer adverse reactions, hypersensitivity reactions (HSRs) to ICM persist, and the incidence of life-threatening events remains similar between the two types [2].

Immediate HSRs occur within one hour of ICM administration and range from mild cutaneous symptoms to life-threatening anaphylaxis [3]. Although rare, with a pooled incidence of 0.01%, severe immediate HSRs are clinically significant. ICM is among the most commonly reported causes of in-hospital drug-induced anaphylaxis [3–8]. Mechanistically, immediate HSRs can arise through immune-mediated (including IgE-mediated) and non–IgE-mediated pathways, involving activation of mast cells/basophils and mediator release, with proposed roles for pathways such as complement activation [9].

Recent studies have implicated genetic factors in ICM-induced HSRs. For instance, HLA-DRB1*15:02 has been associated with ICM-induced anaphylaxis in Korean populations [10], and HLA-B*38:02 and HLA-B*58:01 alleles have shown associations in other ICM-induced immediate HSRs [11]. These findings raise the possibility that ICM-induced HSRs may involve immune-mediated mechanisms. In particular, it has been proposed that certain HLA variants may enhance antigen presentation to T cells, potentially promoting T-cell–dependent B-cell class switching and the production of ICM-specific IgE [12]. However, the underlying mechanisms remain largely unknown, and while HLA associations provide critical clues, they may not fully account for the diverse spectrum of ICM-HSRs.

Whole exome sequencing (WES) enables comprehensive interrogation of protein-coding regions and is possibly an effective approach for identifying both common and rare variants associated with drug response and adverse drug reactions (ADRs) within pharmacogenomics research [13]. In this study, we performed WES to identify genetic variations potentially contributing to ICM-induced immediate HSRs, evaluating candidate single-nucleotide variant (SNV) and gene-level aggregation of variants, and additionally assessing copy number variable regions to provide a comprehensive genetic landscape.

## Materials and methods

### Study participants and data collection

Adult patients (≥18 years) who experienced immediate HSRs after ICM administration between September 2006 and July 2018, were recruited as the case group. Immediate HSRs were defined as a reaction occurring within one hour of ICM exposure. The control group included individuals who had received ICM at least three times without experiencing immediate HSRs. Adverse events such as nausea or chills were not classified as HSRs. A total of 31 patients were enrolled: 20 with ICM-induced immediate HSRs and 11 controls. All participants provided written informed consent, and this study was approved by the Institutional Review Board of Samsung Medical Center (IRB No. 2016-03-105). Data and biospecimens were accessed for research purposes between May 4, 2016, and March 3, 2021, in accordance with IRB approval. All methods were performed in accordance with the relevant guidelines and regulations.

Clinical data were extracted from electronic medical records, including age, sex, comorbidities, type of ICM used, symptoms/signs of HSRs, severity of reactions, number of previous ICM exposures, and history of anaphylaxis. Symptoms were categorized as cutaneous (erythema, urticaria, angioedema, pruritus), respiratory (dyspnea, stridor, wheezing), gastrointestinal (nausea, vomiting, abdominal pain), cardiovascular (chest tightness, diaphoresis, dizziness), and neurological (syncope). The severity of HSRs was classified according to the American College of Radiology guidelines for contrast media [14].

### ICM-induced immediate HSRs and premedication for HSR

Six non-ionic monomeric low-osmolar ICMs were administered intravenously for CT during the study period: iobitridol (Xenetics; Guerbet), iohexol (Omnipaque; GE Healthcare), iomeprol (Iomeron; Bracco), iopamidol (Pamiray; Dongkook Pharm. Co., Ltd.), iopromide (Ultravist; Bayer Healthcare), and ioversol (Optiray; Mallinckrodt PLC). The specific brand used varied by year, based on annual competitive bidding.

Premedication regimens were automatically recommended through the hospital order communication system when physicians ordered contrast-enhanced CT for patients with a prior history of ICM-induced immediate HSRs. Patients received intravenous chlorpheniramine and hydrocortisone 30 minutes before ICM administration. The steroid dose was adjusted at the discretion of the attending physician, based on the severity of the previous reaction.

### Genomic DNA extraction and library preparation for whole exome sequencing

Genomic DNA was extracted from saliva samples collected using Oragene DNA kits (DNA Genotek Inc.), and purified using the QIAamp DNA Mini Kit (QIAGEN), according to the manufacturer’s instructions. DNA concentrations were quantified using a NanoDrop 2000 spectrophotometer (Thermo Fisher Scientific).

Exome libraries were prepared from 100 ng of genomic DNA using the Ion AmpliSeq Library Kit Plus and Ion AmpliSeq Exome RDY Kit (Thermo Fisher Scientific), following the manufacturer’s protocol. Barcoded libraries were generated using the Ion Xpress Barcode Adapter Kit and purified twice with AMPure XP Reagent (Beckman Coulter). Library quality was assessed using an Agilent 2100 Bioanalyzer (Agilent Technologies) and diluted to 100 pM. Libraries were loaded onto an Ion Chef instrument for templating using the Ion 540 Kit-Chef (Thermo Fisher Scientific). Sequencing was performed using the Ion S5XL system (Thermo Fisher Scientific).

### Germline variant calling and preprocessing

Sequencing reads were aligned to the GRCh37 human reference genome using the Torrent Mapping Alignment Program (TMAP, Torrent Suite Software v5.10.0). Binary alignment map (BAM) files were generated for each sample. Germline variants were identified using the Torrent Variant Caller (TVC) with parameters recommended by Thermo Fisher Scientific (S1 Table). Variants were annotated using Ensembl Variant Effect Predictor [15]. Only autosomal variants present in the 1000 Genomes Project [16] or Genome Aggregation Database [17] were included in downstream analyses.

### Single-nucleotide variant analysis

**Fig 1:**
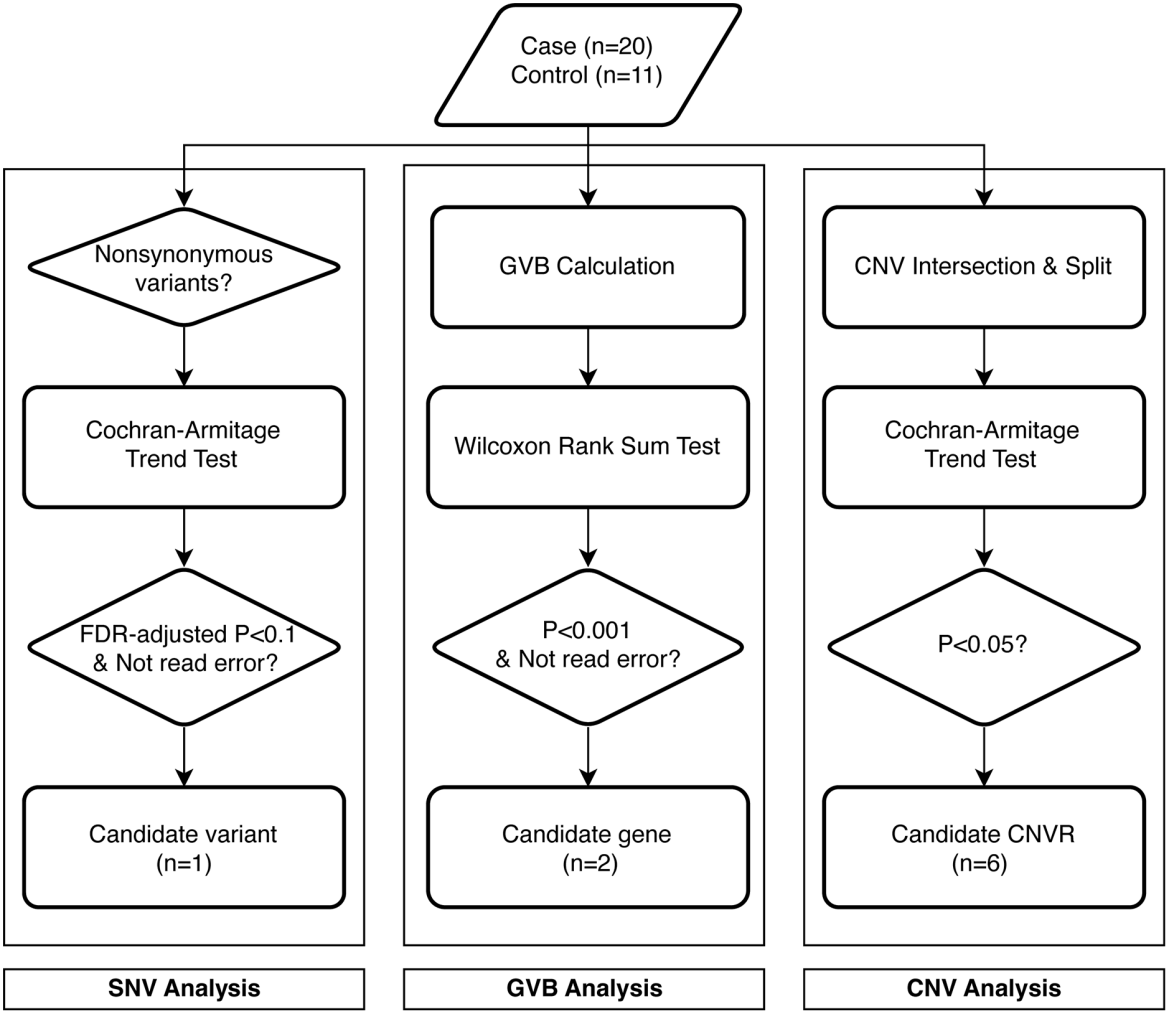
Flowchart for analyses. **Three independent analyses were conducted.** SNV, single-nucleotide variant; GVB, gene-wise variant burden; CNV, copy number variation; CNVR, copy number variation region; FDR, false discovery rate.

Non-synonymous variants were selected, resulting in 8,664 variants for SNV analysis (**Fig 1**). Associations between genotypes and phenotypes were evaluated using the Cochran–Armitage trend test (CATT). A false discovery rate (FDR)-corrected *p*-value < 0.1 was considered significant. Statistically significant variants were manually reviewed in raw sequence data to exclude false positives.

### Gene-wise variant burden analysis

To assess gene-level aggregation of variants, gene-wise variant burden (GVB) scores were calculated for 10,756 genes (**Fig 1**) [18]. The Wilcoxon rank-sum test was used to compare GVB scores between groups. Genes with nominal *p*-values < 0.001 were considered statistically significant. Variants in significant genes were manually reviewed, and genes containing false positives were excluded.

### Detection and analysis for copy number variations

**Fig 2:**
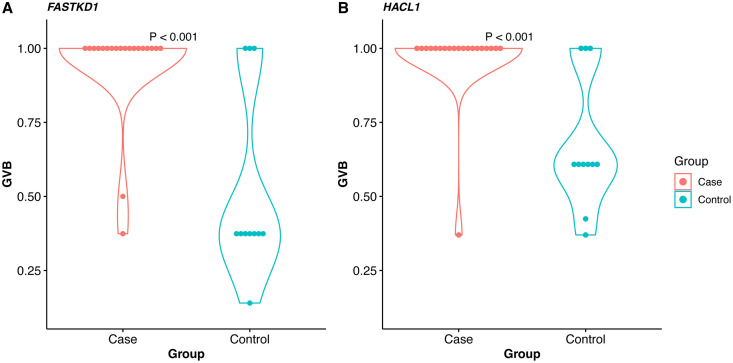
Significant genes in gene-wise variant burden (GVB) analysis. **(A)** *FASTKD1* and **(B)** *HACL1* showed statistically significantly lower GVB in the control group than in the case group (Wilcoxon rank sum test).

CNVs were detected using GATK-gCNV and cn.MOPS, as the combination of multiple tools improves CNV detection [19,20]. Overlapping CNVs (duplication or deletion) from the two tools were identified per patient using the R package GenomicRanges, and CNV regions (CNVRs) were defined using the R package CNTools (S1 Fig). A total of 39 CNVRs were included in downstream analyses. CATT was performed for CNVRs, with a significance threshold of *p* < 0.01 (**Fig 2**).

### Analyses of public data

To assess gene expression in immune cells, transcriptomics data across 18 immune cell types and total PBMCs were obtained from the Human Protein Atlas (HPA) version 23.0 [21]. Cell specificity and expression clusters were also extracted. Conserved regions and protein domains of candidate genes were examined using the UCSC Genome Browser. Correlations between CNVs and gene expression were analyzed using Spearman correlation, based on RNA and CNV data from the Cancer Cell Line Encyclopedia (CCLE) [22].

### Statistical analysis

Categorical variables are expressed as numbers (%), and continuous variables as medians with interquartile ranges (IQR). Comparisons were made using Pearson’s chi-square or Fisher’s exact test for categorical variables and the Mann–Whitney U test for continuous variables. All analyses were conducted using R version 4.1.2.

## Results

### Baseline characteristics of study populations

The clinical characteristics of the study participants are summarized in **Table 1**. The case group included a higher proportion of female patients than the control group (50% vs 9%, *p* = 0.047). Malignancy was the most common comorbidity in both groups, whereas cardiovascular disease was more prevalent in the control group; however, the difference was not statistically significant.

**Table 1:** Baseline characteristics.

Characteristics	Case (*n* = 20)^a^	Control (*n* = 11)^a^	*p*-value
Age, years	56 (49–61)	57 (53–67)	0.270
Sex, Female	10 (50)	1 (9)	0.047
Comorbidities			
Malignancy	18 (90)	10 (91)	1.000
Cardiovascular disease	2 (10)	5 (45)	0.067
Liver diseases	9 (45)	2 (18)	0.241
Allergic disease	3 (15)	2 (18)	1.000
Involved organs during HSRs^b^			
Cutaneous symptoms	17 (85)	N/A	
Cardiovascular symptoms	5 (25)	N/A	
Respiratory symptoms	3 (15)	N/A	
Gastrointestinal symptoms	8 (40)	N/A	
Severity of ICM induced immediate HSR			
Mild	7 (35)	N/A	
Moderate	9 (45)	N/A	
Severe	4 (20)	N/A	
Number of previous exposures to ICM	9 (7-20)	8 (7-11)	0.395
Prior history of ICM induced anaphylaxis	11 (55)	0	0.001

a Values are presented as median (interquartile range) or number (%).

b Some patients showed involvement of more than two organs.

ICM, radiocontrast media; HSR, hypersensitivity reactions; N/A, not applicable.

Cutaneous symptoms were the most common manifestations of ICM-induced immediate HSRs. Severe reactions accompanied by hypoxia, hypotension, or neurologic compromise were observed in 20% of the case group. These patients received antihistamines and intravenous corticosteroids prior to subsequent ICM administration. The number of prior ICM exposures did not differ significantly between the two groups (*p* = 0.219). However, more than half of the patients in the case group had a history of ICM-induced immediate HSRs (*p* = 0.001). The types of ICM administered are listed in S2 Table.

### Genetic variations associated with ICM-induced immediate HSRs

The mean sequencing depth did not differ significantly between the case and control groups (S2 Fig). In the SNV analysis, the FAST kinase domain 1 (*FASTKD1*) gene variant rs12618227 was identified with an FDR-corrected *p* < 0.100 (**Table 2**). In GVB analysis, *FASTKD1* and 2-hydroxyacyl-CoA lyase 1 (*HACL1*) showed significantly lower scores in the control group (nominal *p* < 0.001) (**Fig 2**). Notably, the rs12618227 variant in *FASTKD1*, one of the two *FASTKD1* variants included in the GVB analysis, was also significant in the SNV analysis (S3 Table).

**Table 2:** Significant variant in SNV analysis.

Gene	rsID	Allele^a^	Consequence	AA	Case			Control			Q-value^b^
					WT	HET	HOM	WT	HET	HOM	
*FASTKD1*	rs12618227	C > G	missense	E > Q	19	1	0	3	7	1	0.090

a Reference allele > alternative allele.

b Cochran-Armitage trend test.

*AA,* amino acid; *WT,* wild-type; *HET,* heterozygotes; *HOM,* homozygotes.

CNV analysis revealed five CNVRs in Mucin 12, cell surface associated (*MUC12*) and one CNVR in Signal Regulatory Protein Beta 1 (*SIRPB1*) (S4 Table). A weak but statistically significant correlation was observed between gene copy number and expression levels for these genes in the CCLE database (ρ = 0.16, *p* < 0.001) (S3 Fig).

**Fig 3:**
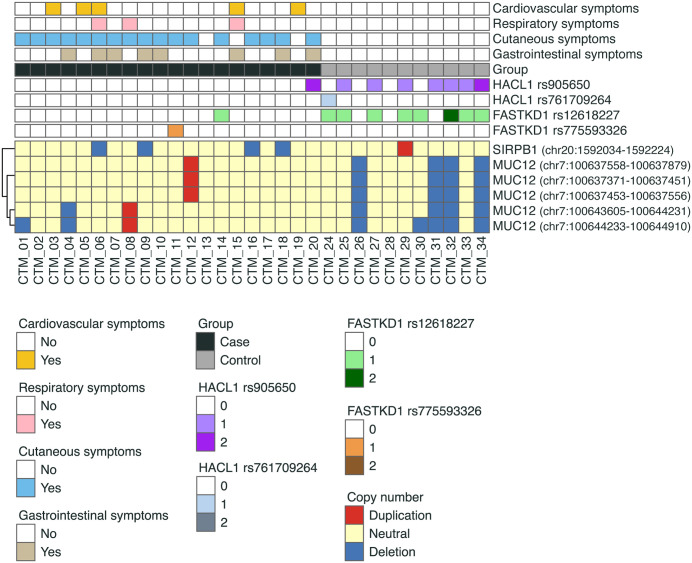
Profiles of genetic variations and ICM-induced HSRs. Heterozygotes or homozygotes of *FASTKD1* and *HACL1* variants were mostly distributed in the control group. The deletion of *SIRPB1* occurred only in the case group, whereas *MUC12* deletion was observed in both groups. 0, 1, 2 in *FASTKD1* and *HALC1* refer to wild-type, heterozygotes, and homozygotes, respectively.

Patient profiles showing significant genetic variations and clinical features of ICM-induced immediate HSRs are presented in **Fig 3**. The alternative alleles of *FASTKD1* rs12618227 and *HACL1* rs905650 were also observed in the control group. Although the CNVR deletion in *SIRPB1* was found exclusively in the case group, CNV event in *MUC12* were found in both groups. All significant genetic variations, except for those in *MUC12*, were located in protein-coding or highly conserved regions, as assessed using the UCSC genome browsers (S4 Fig).

### Expression of cell types in HPA

**Fig 4:**
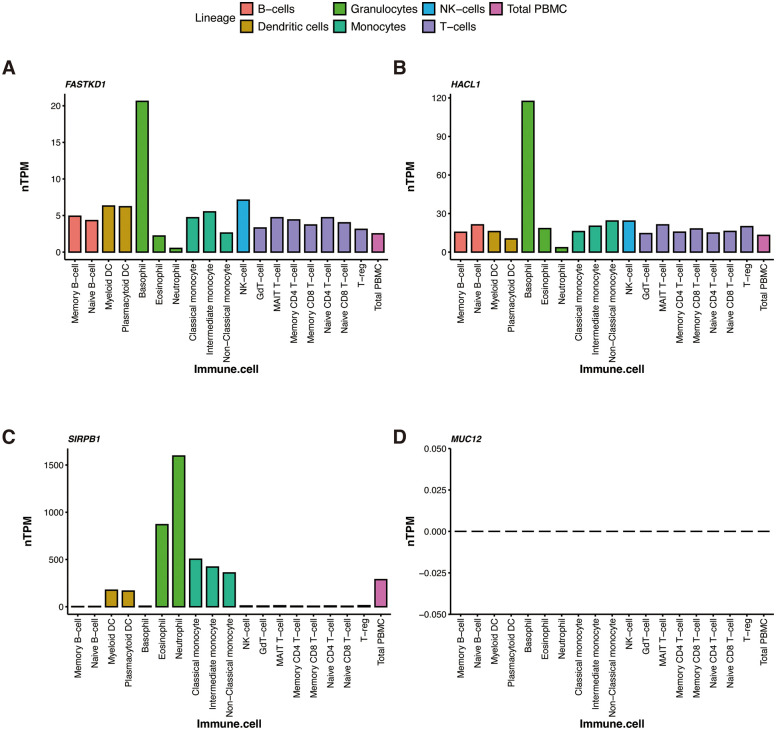
RNA expression levels of candidate genes in immune cells in HPA. **(A)** *FASTKD1* and **(B)** *HACL1* were most expressed in the basophils. **(C)** *SIRPB1* exhibited the highest expression level in the neutrophil. **(D)** *MUC12* was not expressed in all immune cells (nTPM = 0).

The immune cell expression profiles of the four genes from the three analyses are shown in **Fig 4**. Expression of levels of granulocytes was very high in *FASTKD1*, *HACL1*, and *SIRPB1*. *FASTKD1* and *HACL1* showed the highest expression levels in basophils. However, *MUC12* was not expressed in any immune cell (nTPM = 0). At the single-cell level in liver, *SIRPB1* was highly expressed in monocytes, Kupffer cells, and other cells of the mononuclear phagocytic system (S5 Fig).

## Discussion

This study identified genomic variations potentially associated with ICM-induced immediate HSRs. To our knowledge, this is the first WES-based study on ICM-induced immediate HSRs. Previous genomic studies related to adverse reactions to contrast agents have been limited primarily to HLA genotyping. WES offers a powerful approach to detect rare coding variants and heritable factors that may be missed by genome-wide association studies focusing on common variants. It has been successfully applied to elucidate the genetic basis of complex diseases.

*FASTKD1* variant (rs12618227) in ICM-induced immediate HSRs was a significant finding of this study. The variant is most prevalent in East Asians but also occurs in other populations, including Europeans, South Asians, and Americans, with an allele frequency >1% (S3 Table). *FASTKD1* is a mitochondrial protein that downregulates NADH dehydrogenase 3 (ND3) mRNA, a subunit of mitochondrial respiratory complex I [23]. As a result, *FASTKD1* can reduce the activity of the mitochondrial electron transport chain [24]. Mitochondrial damage is typically considered an “off-target” effect of a drug and mitochondrial dysfunction has been implicated in drug-induced toxicities [25], and anaphylaxis is known to cause rapid mitochondrial dysfunction and oxidative damage in cardiac tissue [26]. Although the sample size was small and clinical confounders were not fully accounted due to the limitations of the available medical records, we observed that the frequency of rs12618227 in *FASTKD1* was approximately 14.5 times higher in the control group than in the case group. This suggests a possible protective role in ICM-induced hypersensitivity reactions (S3 Table). Similarly, *FASTKD1* showed low GVB scores in the control group (**Fig 2A**). The GVB score was obtained by integrating relatively conserved variants (SIFT ≤ 0.7) [18]. Therefore, genes with low GVB scores were likely to be functional. The fact that it was low in the control group suggests that the gene may have tolerance functions in HSRs. We acknowledge that this finding should be interpreted with caution. Given the sample size and lack of detailed phenotype stratification, our results are exploratory in nature and require validation in larger, clinically well-characterized cohorts.

Similar to *FASTKD1*, *HACL1* also showed low GVB scores in the control group (**Fig 2B**). *HACL1* is involved in peroxisomal fatty acid α-oxidation and arachidonic acid metabolism [27]. In mammals, peroxisomes play an indispensable role in modulating diverse physiological and pathological processes, including inflammation, innate immunity, and cell fate transitions, by serving as signaling platforms and participating in the metabolism of reactive oxygen and reactive nitrogen species [28]. It has become increasingly clear that defects in peroxisome biogenesis [29], peroxisomal fatty acid metabolism [30], or peroxisomal antioxidant capacity [31] negatively impact mitochondrial function. Thus, *HACL1* may contribute to immune responses and mitochondrial stability, both of which are relevant in the context of ICM-induced HSRs.

One significant CNVR was identified in *SIRPB1* gene (S4 Table). CNVR includes immunoglobulin domains and *SIRPB1* is associated with the innate immune system, which plays a role in promoting phagocytosis by macrophages and facilitating the migration of neutrophils to regulate the inflammatory response [9,32]. *SIRPB1* is also involved in secretory granule exocytosis (Reactome pathway R-HSA-6804801) [33]. Although *MUC12* was statistically significant in the CNV analysis, it appeared to have limited functional relevance and evolutionary conservation (S4 Fig).

Data from the HPA indicated that *FASTKD1* and *HACL1* are highly expressed in basophils, while *SIRPB1* is enriched in neutrophils (**Fig 4**). Basophils and mast cells play central roles in immunoglobulin (Ig)E-mediated immediate drug hypersensitivity reactions through high-affinity IgE receptors (FcεRI) activation, which leads to the release of histamine and tryptase [34]. A recent study showed that ICM increase the degranulation of mast cells and basophils, resulting in the release of inflammatory mediators [9,35,36].

It is also noteworthy that *SIRPB1* has enhanced expression levels in neutrophils. Drug-specific IgG complexes can activate neutrophils *ex vivo* and increase the number of circulating activated neutrophils in patients with anaphylaxis, potentially triggering anaphylactic symptoms through the rapid release of potent lipid and protein mediators and the expulsion of neutrophil extracellular traps (NETs) via NETosis, which involves the expulsion of nuclear and mitochondrial DNA [37,38]. Moreover, according to HPA, *SIRPB1* shows “*group enriched”* single cell specificity in monocytes, Kupffer cells, dendritic cells, macrophages, and Hofbauer cells. These cells are included in the human mononuclear phagocytic system (MPS), a critical regulator of innate and adaptive immune responses [39]. In HPA liver single-cell dataset, *SIRPB1* shows its highest expression in Kupffer cells (S5 Fig). Kupffer cells in the liver recognize particulate drug carriers delivered intravenously as foreign agents within the MPS, resulting in their rapid clearance from the circulation [40]. However, drug delivery systems targeting the MPS render important host defense systems susceptible to toxic effects owing to the accumulation of drug [41]. Kupffer cells, in particular, are involved in the clearance of intravenously administered substances, including ICM [42]. Additionally, ICM can impair granulocyte and monocyte phagocytosis, potentially contributing to HSRs [43].

*SIRPB1* may also be relevant in IgG-mediated anaphylaxis, which has been demonstrated in murine models and involves FcγRIII-mediated activation of neutrophils, macrophages, and basophils, resulting in the release of platelet-activating factor and NETs [44]. Given the high expression of *FASTKD1*, *HACL1*, and *SIRPB1* in these immune cells, the findings may have broader implications for both IgE- and IgG-mediated mechanisms of ICM-induced anaphylaxis.

Of note, this study used saliva-derived DNA for WES, which demonstrated comparable quality to blood-derived DNA for germline variant analysis [45], thereby providing a non-invasive and practical alternative to blood collection. Nonetheless, future validation studies incorporating blood samples and cytokine profiling are warranted to further elucidate the mechanisms of ICM-induced HSRs.

This study has several limitations. First, the limited sample size may have constrained the statistical power for novel gene discovery. Second, liver disease was more prevalent among cases than controls, although this difference did not reach statistical significance (*p* = 0.241). While hepatic dysfunction could theoretically influence the metabolism and clearance of iodinated contrast media, potentially modifying susceptibility to adverse reactions, this hypothesis requires further investigation. Future studies in larger, independent cohorts are warranted to more rigorously account for liver disease status and its severity. Third, different ICM types were used between groups due to changes in hospital formularies. However, a recent meta-analysis reported no significant differences in acute reaction rates among nonionic ICMs after adjusting for confounders [46]. Lastly, confirmatory laboratory tests such as serum tryptase or skin testing were not available at the study period.

## Conclusions

This study identified *FASTKD1*, *HACL1*, and *SIRPB1* as candidate genes potentially involved in ICM-induced immediate HSRs. These findings may contribute to future efforts in biomarker discovery and the development of precision medicine approaches in radiologic practice. Larger prospective studies are warranted to validate and expand upon these results.

## Supporting information

S1 Fig Intersection and split of CNVs. (**A**) Intersection of copy number variations derived from multiple callers per patient. (**B**) Splitting intersection CNVs into CNVRs between patients. *CNV* copy number variations, *CNVR* copy number variations regions.
(PNG)

S2 Fig Mean depth for sequencing reads in two groups. Wilcoxon rank sum test was performed on the read depth between two groups (*p* = 0.451).
(JPG)

S3 Fig Correlation between RNA expression and copy number of genes in CCLE. (**A**) *MUC12* and (**B**) *SIRPB1* showed significant correlation in their copy number and expression.
(PNG)

S4 Fig Significant genetic variations in UCSC genome browser. Red vertical lines indicate significant genetic variations. (**A**) rs775593326 in *FASTKD1*. (**B**) rs12618227 in *FASTKD1*. (**C**) rs761709264 and rs905650 in *HACL1*. (**D**) CNVR in *SIRPB1*. (**E**) CNVRs in *MUC12*.
(PNG)

S5 Fig Single-cell type expression of *SIRPB1* in liver from HPA. UMAP projection of liver cells grouped into clusters (c-0–19) annotated by representative cell types.
(PNG)

S1 Table Parameters for germline variant calling in TVC. (DOCX)

S2 Table Type of ICM. (DOCX)

S3 Table Variants and genes in SNV or GVB analysis. (DOCX)

S4 Table Significant CNVRs. (DOCX)
