# Correction: Genetic variations associated with immediate hypersensitivity reactions to iodinated contrast media: A whole exome sequencing study

**DOI:** 10.1371/journal.pone.0348213

**Published:** 2026-04-27

**Authors:** The PLOS One Staff

S5 Fig was uploaded incorrectly. Please see the correct S5 Fig here.

The publisher apologizes for the error.

## Supporting information

S5 Fig Single-cell type expression of SIRPB1 in liver from HPA. UMAP projection of liver cells grouped into clusters (c-0–19) annotated by representative cell types.
(PNG)
