# Supplementary figures and images for: Genetic variations associated with immediate hypersensitivity reactions to iodinated contrast media: A whole exome sequencing study
Source: PLoS One. 2026 Mar 26;21(3):e0345313. doi: 10.1371/journal.pone.0345313 (PMC13020841; doi:10.1371/journal.pone.0345313)

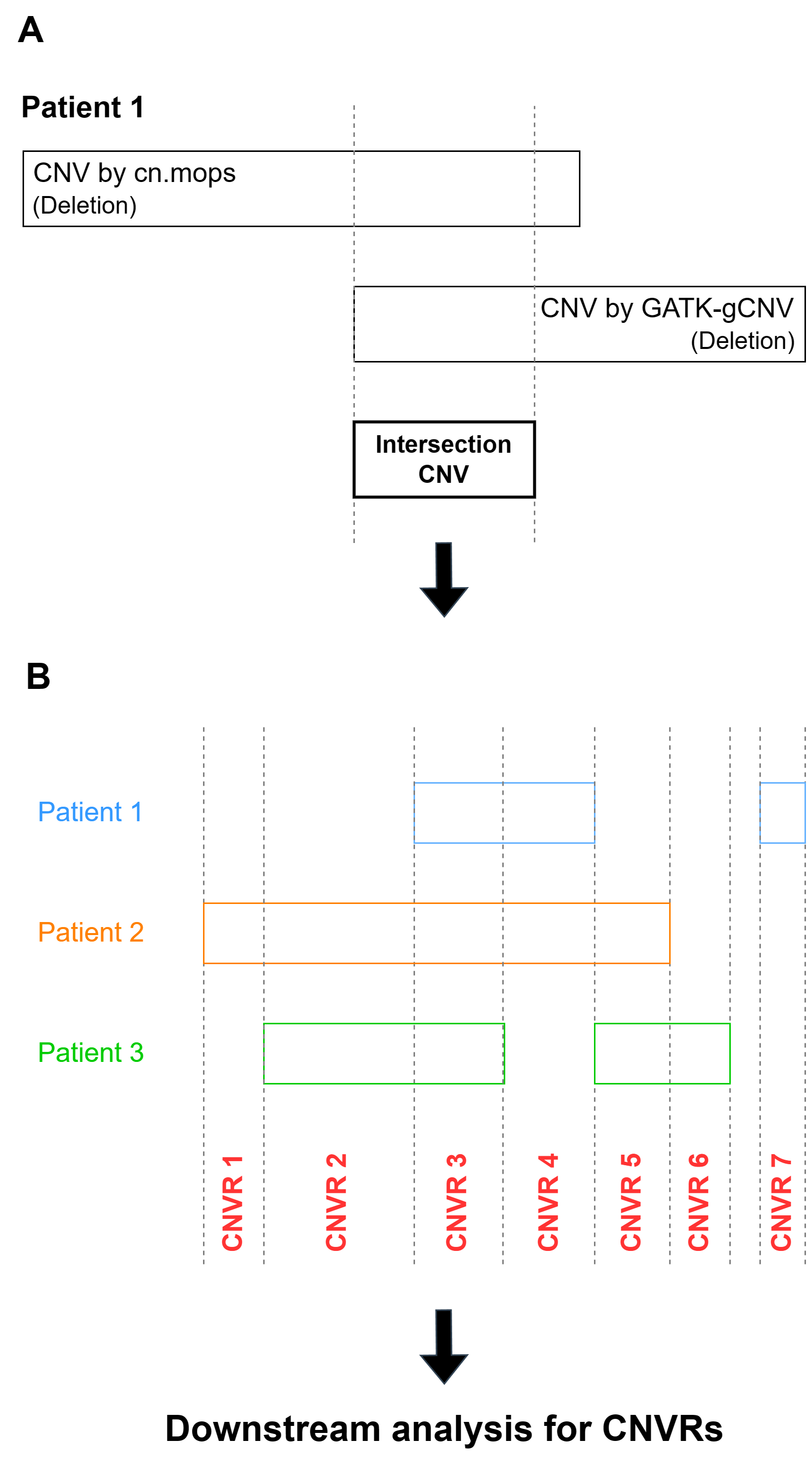

Supplement: S1 Fig — (A) Intersection of copy number variations derived from multiple callers per patient. (B) Splitting intersection CNVs into CNVRs between patients. CNV copy number variations, CNVR copy number variations regions. (PNG) [file pone.0345313.s001.png]

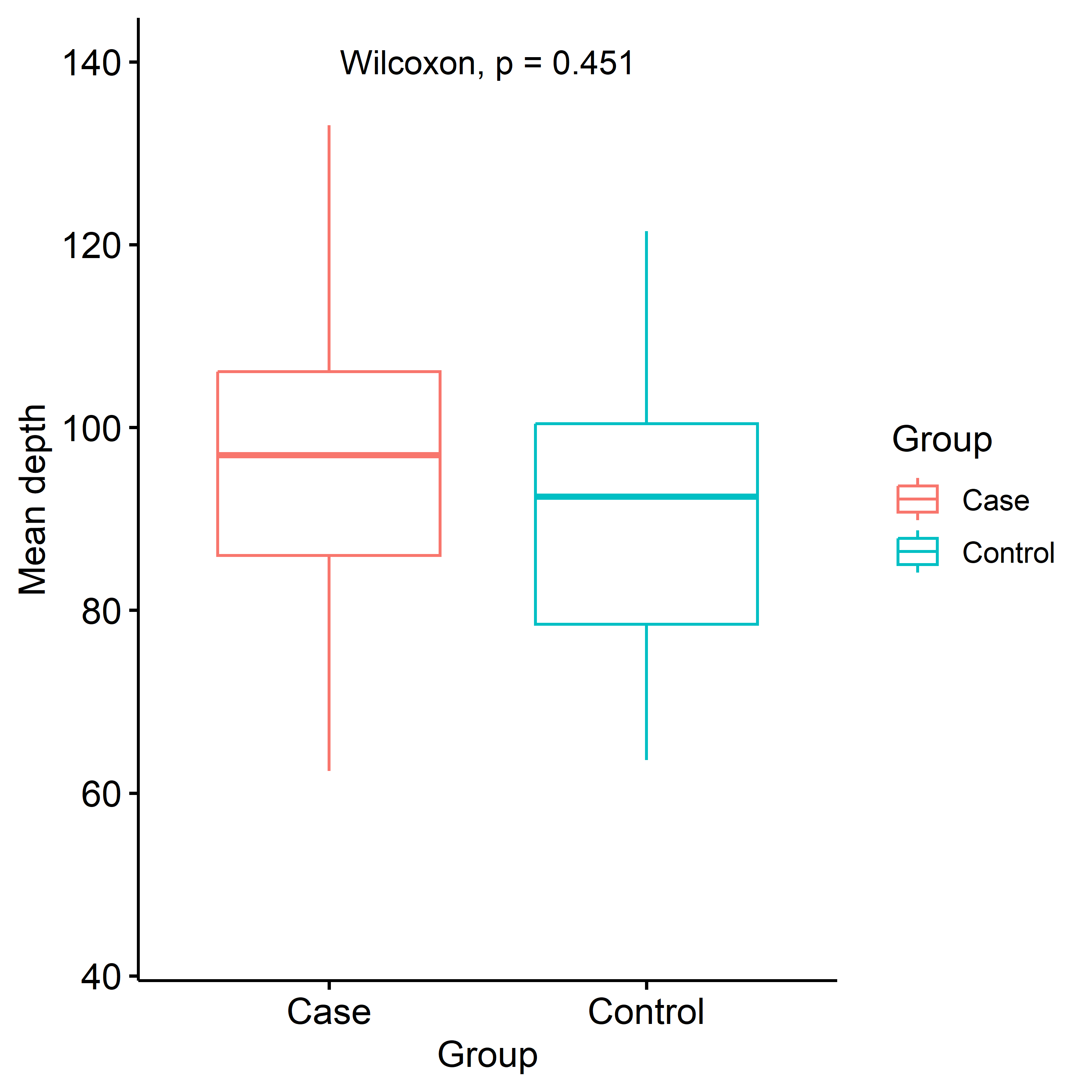

Supplement: S2 Fig — Wilcoxon rank sum test was performed on the read depth between two groups (p = 0.451). (JPG) [file pone.0345313.s002.jpg]

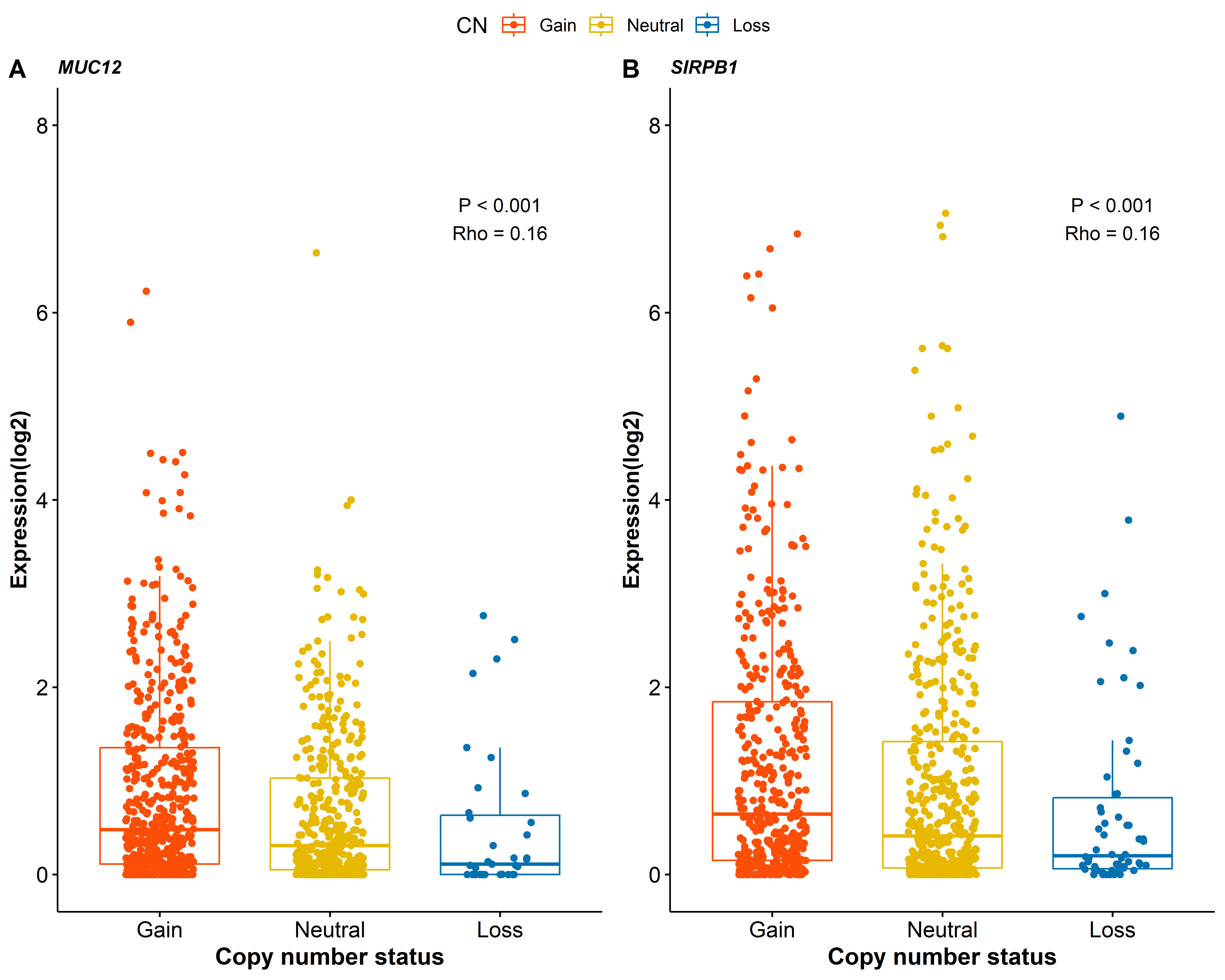

Supplement: S3 Fig — (A) MUC12 and (B) SIRPB1 showed significant correlation in their copy number and expression. (PNG) [file pone.0345313.s003.png]

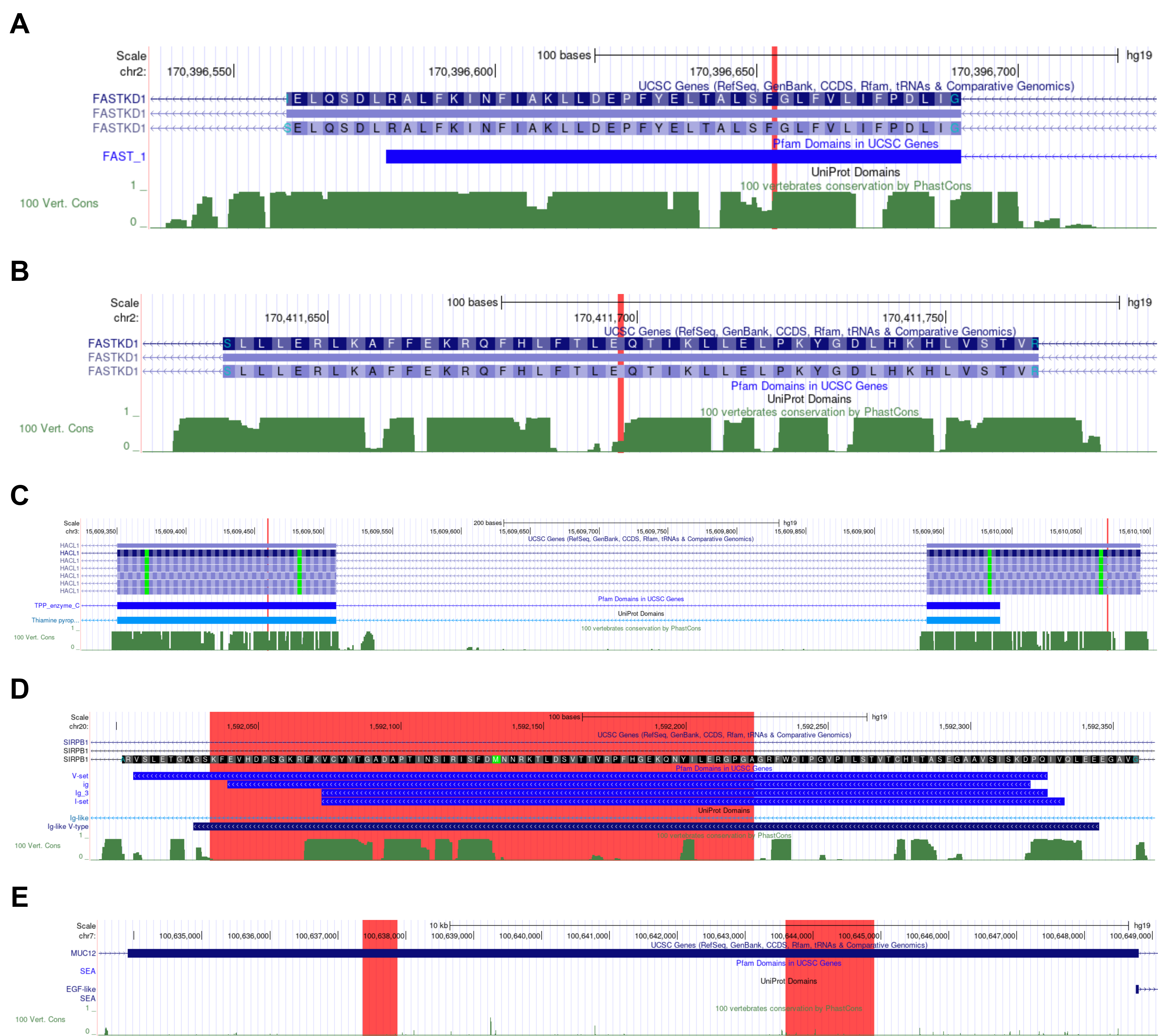

Supplement: S4 Fig — Red vertical lines indicate significant genetic variations. (A) rs775593326 in FASTKD1. (B) rs12618227 in FASTKD1. (C) rs761709264 and rs905650 in HACL1. (D) CNVR in SIRPB1. (E) CNVRs in MUC12. (PNG) [file pone.0345313.s004.png]
