# Supplementary material for: Genetic variations associated with immediate hypersensitivity reactions to iodinated contrast media: A whole exome sequencing study
Source: PLoS One. 2026 Mar 26;21(3):e0345313. doi: 10.1371/journal.pone.0345313 (PMC13020841; doi:10.1371/journal.pone.0345313)
Supplement: S1 Table — (DOCX) [file pone.0345313.s006.docx]

| **Parameter** | **Value** | **Recommended values** |
| --- | --- | --- |
| snp_min_allele_freq | 0.2 | 0.01 - 0.2 |
| snp_min_cov_each_strand | 3 | >= 3 |
| snp_min_coverage | 10 | 5 - 20 |
| snp_min_variant_score | 15 | >= 10 |
| snp_strand_bias | 0.95 | 0.95 |
| indel_min_allele_freq | 0.2 | 0.05 - 0.2 |
| indel_min_cov_each_strand | 6 | >= 3 |
| indel_min_coverage | 30 | 15 - 30 |
| indel_min_variant_score | 30 | >= 10 |
| indel_strand_bias | 0.85 | 0.85 |

**S1 Table. Parameters for germline variant calling in TVC**

*TVC*, Torrent Variant Caller
