# Supplementary material for: Genetic variations associated with immediate hypersensitivity reactions to iodinated contrast media: A whole exome sequencing study
Source: PLoS One. 2026 Mar 26;21(3):e0345313. doi: 10.1371/journal.pone.0345313 (PMC13020841; doi:10.1371/journal.pone.0345313)
Supplement: S2 Table — (DOCX) [file pone.0345313.s007.docx]

**S2 Table. Type of ICM**

| **Characteristics** | **Case (*n* = 20)** | **Control (*n* = 11)** |
| --- | --- | --- |
| Types of ICMs |  |  |
| Iopromide | 20 (100) | 0 |
| Iobitridol | 0 | 4 (36) |
| Iohexol | 0 | 2 (18) |
| Iomeprole | 0 | 2 (18) |
| Ioversol | 0 | 2 (18) |
| Iopamidol | 0 | 1 (9) |

*ICM*, iodinated contrast media
