# Supplementary material for: Genetic variations associated with immediate hypersensitivity reactions to iodinated contrast media: A whole exome sequencing study
Source: PLoS One. 2026 Mar 26;21(3):e0345313. doi: 10.1371/journal.pone.0345313 (PMC13020841; doi:10.1371/journal.pone.0345313)
Supplement: S3 Table — (DOCX) [file pone.0345313.s008.docx]

**S3 Table. Variants and genes in single variant or GVB analysis**

| **Gene** | **rsID** | **Allele**^a^ | **Consequence** | **Case** | |  | **Control** | |  | **1KGP**^b^ | | | | | |  |
| --- | --- | --- | --- | --- | --- | --- | --- | --- | --- | --- | --- | --- | --- | --- | --- | --- |
|  |  |  |  | **Non-carrier** | **Carrier**^c^ |  | **Non-carrier** | **Carrier** |  | **EAS** | **SAS** | **EUR** | **AMR** | **AFR** |  |  |
| *FASTKD1* | rs12618227 | C>G | missense | 0.950 | 0.050 |  | 0.273 | 0.727 |  | 0.202 | 0.055 | 0.056 | 0.017 | 0.002 |  |  |
|  | rs775593326 | A>G | missense | 0.950 | 0.050 |  | 1 | 0 |  | 0 | 0 | 0 | 0 | 0 |  |  |
| *HACL1* | rs905650 | C>T | synonymous | 0.950 | 0.050 |  | 0.364 | 0.636 |  | 0.310 | 0.235 | 0.231 | 0.127 | 0.130 |  |  |
|  | rs761709264 | T>C | missense | 1 | 0 |  | 0.909 | 0.091 |  | 0 | 0 | 0 | 0 | 0 |  |  |
| ^a^Reference allele > alternative allele  ^b^The 1000 Genomes Project (1KGP)-Allele carrier frequency by populations in 1000 Genomes Project  ^c^Carrier refers to the frequency of people with an alternative allele, and non-carrier refers to the frequency of people with a reference allele  *GVB*, gene-wise variant burden; *EAS,* East Asian; *SAS*, South Asian; *EUR*, European; *AMR*, American; *AFR*, African | | | | | | | | | | | | | | | | |
