# Supplementary material for: Genetic variations associated with immediate hypersensitivity reactions to iodinated contrast media: A whole exome sequencing study
Source: PLoS One. 2026 Mar 26;21(3):e0345313. doi: 10.1371/journal.pone.0345313 (PMC13020841; doi:10.1371/journal.pone.0345313)
Supplement: S4 Table — (DOCX) [file pone.0345313.s009.docx]

**S4 Table. Significant CNVRs**

| **Gene** | **Chromosome** | **Start** | **End** | **Width** | ***p*-value^*^** |
| --- | --- | --- | --- | --- | --- |
| *MUC12* | 7 | 100637371 | 100637451 | 82 | 0.005 |
| *MUC12* | 7 | 100637453 | 100637556 | 105 | 0.005 |
| *MUC12* | 7 | 100637558 | 100637879 | 323 | 0.005 |
| *MUC12* | 7 | 100643605 | 100644231 | 628 | 0.022 |
| *MUC12* | 7 | 100644233 | 100644910 | 679 | 0.022 |
| *SIRPB1* | 20 | 1592034 | 1592224 | 192 | 0.047 |

*CNVR* copy number variation region

^*^Nominal *p*-value from Cochran-Armitage trend test
