# Supplementary figures and images for: Correction: Genetic variations associated with immediate hypersensitivity reactions to iodinated contrast media: A whole exome sequencing study
Source: PLoS One. 2026 Apr 27;21(4):e0348213. doi: 10.1371/journal.pone.0348213 (PMC13119867; doi:10.1371/journal.pone.0348213)

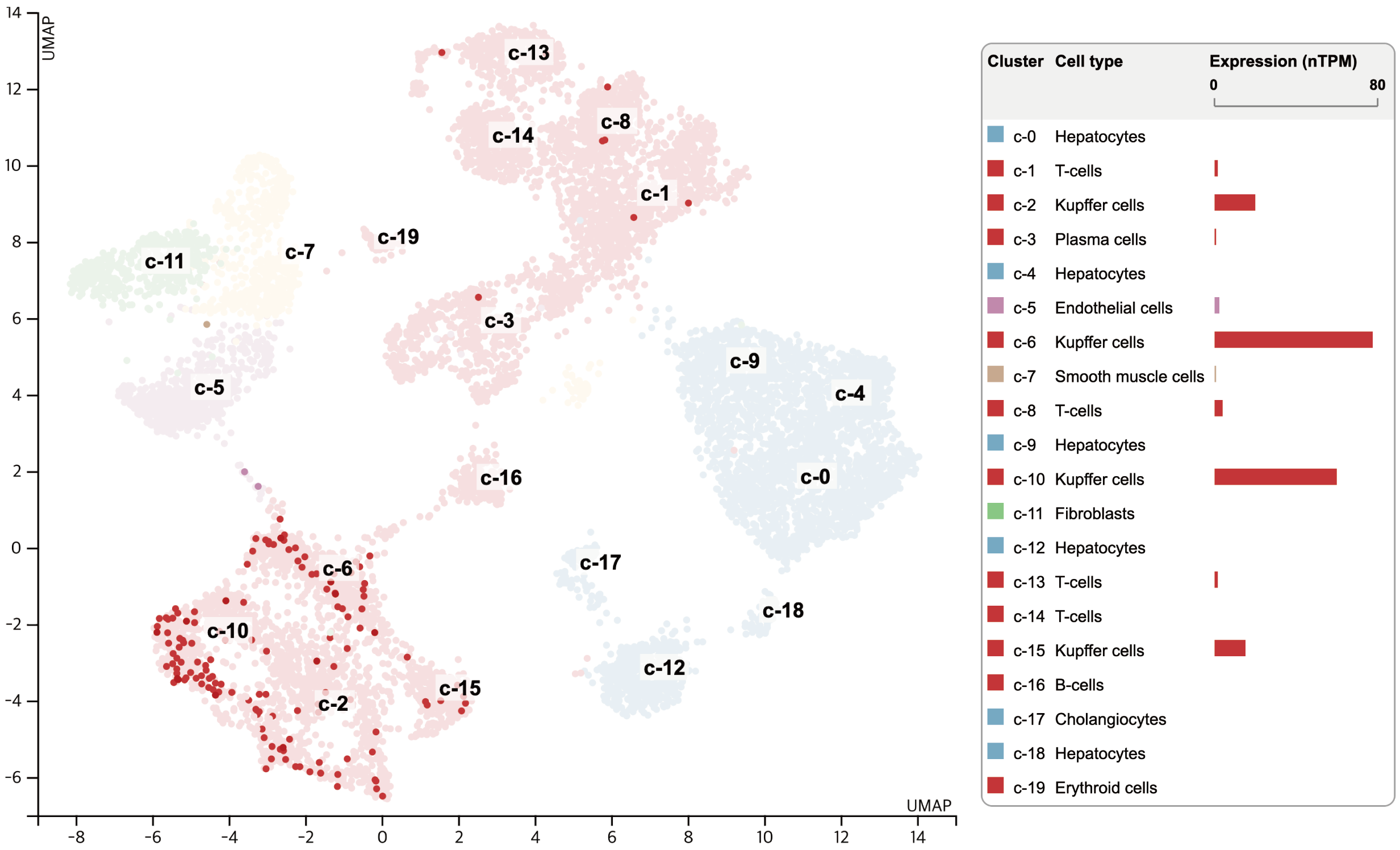

Supplement: S5 Fig — UMAP projection of liver cells grouped into clusters (c-0–19) annotated by representative cell types. (PNG) [file pone.0348213.s001.png]
